# Factors Affecting Embryo Recovery Rate, Quality, and Diameter in Andalusian Donkey Jennies

**DOI:** 10.3390/ani10111967

**Published:** 2020-10-26

**Authors:** J. Dorado, M. Bottrel, I. Ortiz, M. Díaz-Jiménez, B. Pereira, C. Consuegra, J. J. Carrasco, V. Gómez-Arrones, A. Domingo, M. Hidalgo

**Affiliations:** 1 Veterinary Reproduction Group, Department of Medicine and Animal Surgery, Faculty of Veterinary Medicine, University of Cordoba, 14071 Cordoba, Spain; ma.bottrel@gmail.com (M.B.); isabel.ortiz.vet@gmail.com (I.O.); mariadijim@gmail.com (M.D.-J.); blasypereiraaguilar@gmail.com (B.P.); mtc93vet@gmail.com (C.C.); mhidalgo@uco.es (M.H.); 2 Equine Reproduction Center, Centro de Selección y Reproducción Animal, (CENSYRA-Extremadura Government), 06007 Badajoz, Spain; juanjesus.carrasco@juntaex.es (J.J.C.); vanearrones@gmail.com (V.G.-A.); adomingomontes@gmail.com (A.D.)

**Keywords:** donkey, embryo transfer, embryo recovery rate, embryo quality

## Abstract

**Simple Summary:**

Embryo transfer has been successfully used for the conservation of equine endangered species, but a number of factors may affect the outcome of these techniques in mares. However, only a few studies have evaluated these factors in donkeys. The present study was conducted to determine which factors affect the recovery rate, morphological quality, and diameter in embryos from Andalusian donkey jennies. According to our results, the factors affecting embryo recovery rate were donor jenny, donor age, successive cycle within donor, number of flushings, and jack. Day of flushing and number of flushings had an effect on embryo diameter, whereas donor jenny and day of flushing had an effect on embryo quality. The knowledge of these factors is crucial to achieve a higher efficiency of embryo transfer in endangered donkey breeds.

**Abstract:**

Embryo transfer and the vitrification of embryos could be used for the conservation and recovery of endangered donkey breeds. It is important to develop techniques that optimize recovery rates and the cryotolerance of donkey embryos. This study evaluates factors affecting the recovery rate, quality, and diameter of embryos obtained from donor jennies as a starting point for the use of vitrification and embryo transfer in the conservation of the Andalusian donkey. A total of 100 embryos were recovered out of 124 estrous cycles (80.6%). The donor jenny affected the rates of positive flushings (PFR; *p* = 0.040) and embryo recovery (ERR; *p* < 0.05) as well as embryo quality (*p* = 0.004). ERR was also affected by the number of flushings (*p* < 0.001), donor age (*p* < 0.05), successive cycle within donor (*p* < 0.001), and jacks (*p* < 0.05). Number of flushings (*p* < 0.001) and jack (*p* < 0.05) had a significant effect on PFR, whereas the day of flushing influenced the developmental stage (*p* < 0.001), embryo quality (*p* < 0.05), and diameter of embryos (*p* < 0.001). The number of flushings significantly influenced the diameter (*p* = 0.038) and embryo developmental stage (*p* = 0.001), whereas the developmental stage was statistically different between herds (*p* = 0.020). The factors influencing the success of this assisted reproductive technique were donor jenny, donor age, successive cycle within donor, day of flushing, number of flushings, and jack. The identification of these key points is crucial to achieve a higher efficiency of embryo transfer and vitrification processes, before considering their application in the conservation of endangered donkey breeds.

## 1. Introduction

In the past, domestic donkeys (*Equus africanus asinus*) were used as pack animals in agricultural activities, commerce, and militia [1,2], mainly due to their easy care, their resistance to diseases, and their physical resistance [3]. However, the mechanization of agriculture in Europe together with the consequent sharp decrease in mule breeding caused a drastic reduction of the donkey population [4,5,6,7]. Currently, all Spanish donkey breeds (Andaluza, Catalana, Balear, Majorera, Asno de las Encartaciones, and Zamorano-Leonés) are considered endangered (Real Decreto 2129/2008, regulation of the National Catalogue of Endangered Species). Although the population size of the Andalusian donkey has increased to 839 animals in 2018, only 14 females were pure breed. Moreover, the number of herds across Spain has decreased (163 herds), and the average herd size is five heads [8], thereby increasing the possibility of mating of related animals. Considering the contribution of donkeys to biodiversity [9], milk and meat products production [3,10], or pet therapy [2], strategies for the preservation of the genetic pool of donkey breeds and for the maintenance of the genetic heterozygosis of equine endangered species is highly advisable.

The conservation of endangered species is an excellent opportunity for applying assisted reproductive technologies such as embryo transfer, embryo cryopreservation, and germplasm cryobanking. Embryo transfer (ET) has been successfully used for the conservation of equine endangered species such as Przewalski´s horses (*Equus przewalskii*) [11], and numerous studies have been conducted in the past decades to investigate the suitability and efficiency of equine ET [12]. Together with this technique, the cryopreservation of embryos and their storage in embryo banks offer several advantages to the preservation and management of equine endangered species [13,14]. However, in donkeys, the studies on both procedures are scarce and recent [15,16,17,18,19,20,21].

It is known that some factors may affect the embryo recovery rate and embryo diameter and morphological quality in mares, including the day of flushing, number of ovulations, age of the donor, and quality of semen [22], and that morphological embryo quality has a major effect on pregnancy rates [13]. Other factors such as the size and age of embryos and storage of embryos may also affect pregnancy rates after ET in horses [23,24]. In addition, previous studies have demonstrated that early-stage horse embryos (<300 μm) show better survival rates after cryopreservation than large embryos collected at a later day [25,26]. In donkeys, only a few studies have been conducted, and the results could not prove the influence of embryo quality and age on embryo recovery rate [16,17]. Similarly, no effect on embryo recovery rate and quality was observed by Pérez-Marín et al. [20]

The aim of the present study was to determine which factors affect the recovery rate, morphological quality, and diameter in embryos from Andalusian donkey jennies as a prerequisite to improve the success of both embryo transfer and cryopreservation in this endangered donkey breed.

## 2. Materials and Methods

### 2.1. Experimental Animals and Study Location

All animal procedures were approved by the Ethical Committee for Animal Experimentation of the University of Cordoba (no. 31/08/2017/105) and are in accordance with Spanish laws for animal welfare and experimentation (Real Decreto 53/2013).

From February to December of three consecutive years (2015–2017), a total of twenty-six healthy Andalusian jennies (3–13 years old), of known fertility, served as embryo donors, and eight Andalusian jacks (6–9 years old) known to be fertile were used to mate the donors. To assess the effect of the age, the donor jennies were divided into three categories: ≤3 (*n* = 5), 4–9 (*n* = 17), and ≥10 years old (*n* = 5). 

General health and reproductive history were recorded, and jennies were submitted to a general and reproductive physical examination [23]. Donors were housed, monitored, mated, and flushed in three different herds: the Equine Center for Assisted Reproduction of the Centro de Selección y Reproducción Animal (CENSYRA, Badajoz, Spain), the Centro Rural Malpica (Palma del Río, Cordoba, Spain) or the Centro de Medicina Deportiva Equina (CEMEDE, Cordoba, Spain). The jennies were housed in paddocks, the jacks were housed in stalls, and they were fed with hay, barley, and water ad libitum.

### 2.2. Oestrus Synchronization and Mating

Ovarian activity was evaluated by transrectal ultrasonography (Aloka SSD 500, ALOKA Co. Ltd., Tokyo, Japan) on a biweekly schedule during diestrus and daily during oestrus until ovulation (Day 0 = day of ovulation). Estrus was induced with one intramuscular injection of 5.25 mg luprostiol (Prosolvin^®^, Virbac, Barcelona, Spain) in the presence of corpus luteum. Donor jennys received human chorionic gonadotropin (hCG; 1500 IU, intramuscularly; Veterin Corion^®^, Divasa-Farmavic S.A., Barcelona, Spain) to induce ovulation when a follicle of 35–40 mm was detected. Next day, donor jennies were bred by live cover every other day until ovulation.

### 2.3. Embryo Recovery and Evaluation

**Figure 1:**
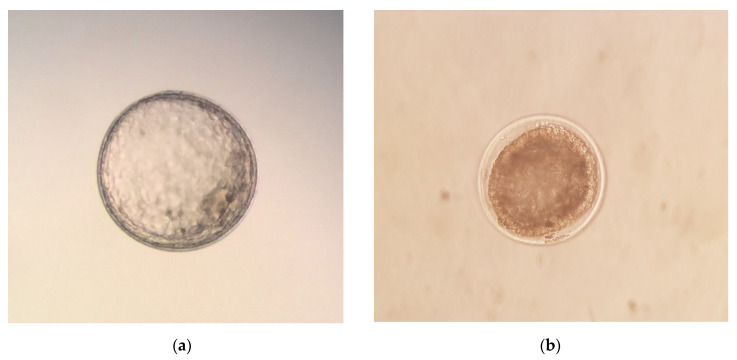

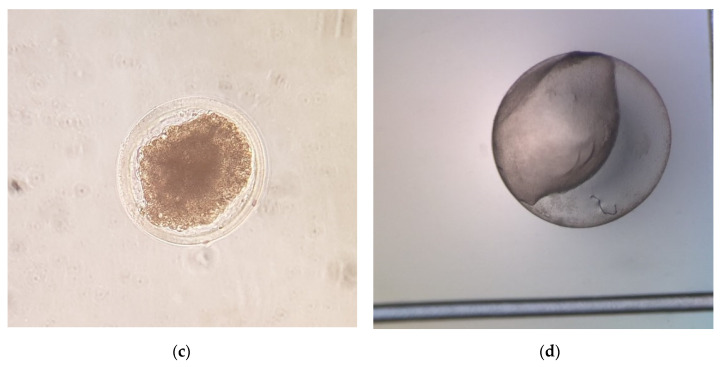
Donkey embryos of various developmental stages and quality grades. (**a**) Expanded blastocyst stage embryo, Grade 1. Note blastocoele cavity and distinct inner cell mass. The zona pellucida has been shed, and the capsule is surrounding the embryo. No morphologic abnormalities are present in this embryo; (**b**) Blastocyst stage embryo, Grade 2. Note the distinct blastomere cells around the edge of the embryo and the capsule. A blastocoele cavity is just beginning to form within the center of the embryo. Note minor imperfections, such as a few extruded cells, occasional discolored cells, and slight shrinkage of trophoblast from zona pellucida; (**c**) Early blastocyst stage embryo, Grade 3. Note thick zona pellucida and capsule. Note moderate level of imperfections, such as a high proportion of extruded cells, discoloration of remaining cell mass, and moderate shrinkage of trophoblast from zona pellucida; (**d**) Expanded blastocyst stage embryo, Grade 4. Note complete collapse of the blastocoele.

Six to nine days after ovulation, donor jennies were flushed 3 times with a total of 3 L of Lactated Ringer´s solution (B. Braun VetCare S.A., Rubí, Spain), as described by Camillo et al. [17] for donkeys. After the flushing, luprostiol was administered to donors to induce luteolysis. Recovered embryos were evaluated for developmental stage (morula, early blastocyst, blastocyst, or expanded blastocyst) and morphological quality, and they were graded on a scale of 1–4 [27], 1 being excellent, 2 being good, 3 being fair, and 4 being poor, degenerate, or dead (Figure 1). After the quality evaluation, the embryos were washed ten times in Syngro^®^ holding (Bioniche Animal Health, Washington, DC, USA), as previously described [16]. The diameter of the embryos was measured under bright field conditions (SZ51 Olympus optical, Tokyo, Japan) using an ocular micrometer (scale of 1 mm/100), as previously described [28].

### 2.4. Statistical Analysis

Descriptive statistical analysis was performed, presenting the qualitative variables as frequencies and percentages, and quantitative as a mean ± standard error of the mean (SEM). The effects of the year in which the study was performed (1–3), season (winter: December 22–March 20; spring: March 21–June 20; summer: June 21–September 22; autumn: September 23–December 21), photoperiod (positive: March–October; negative: November–February), herd (1–3), days of flushing for embryo recovery (6–9), number of flushings (1–3), donor (25 jennies), donor age (≤3; 4–9; ≥10 years old), parity (nulliparous vs. multiparous), successive cycle within donor (1–5; 6–14; 15–23), number of ovulations per cycle (single vs. double), and jack (8 donkeys) on positive uterine flushing rates (PFR; flushing where at least one embryo was recovered), embryo recovery rate (ERR; embryos recovered per cycle), and on ovulation rate (OR; number of ovulations per cycles) were analyzed by the Chi-square test and by the Kruskal–Wallis one-way ANOVA, respectively. When the effect was statistically significant, post-hoc multiple comparisons were made using Chi-square tests for categorical variables and Mann–Whitney U tests for continuous variables. 

To evaluate the effects of single factors (year, season, photoperiod, herd, day of flushing, number of flushings, donor, donor age, parity, successive cycle within donor, number of ovulations, and jack) on embryo quality (Grade 1–4), diameter (µm), and developmental stage (morula, early blastocyst, blastocyst, expanded blastocyst) the Kruskal–Wallis one-way ANOVA was performed. Mean values were compared by Duncan’s test.

All analyses were performed using the statistical package SPSS v15.0 (IBM Spain, Madrid, Spain). Differences were considered statistically significant when *p* < 0.05.

## 3. Results

The average ovulation rate in donor jennies is shown in Table 1. The average rate per jenny was 1.26 ± 0.04 and varied significantly among jennies (*p* = 0.01). Results of statistical analysis also showed differences among herds (*p* = 0.031), while no differences (*p* > 0.05) were observed among donor age categories, years, photoperiods, and seasons (Table 1). The single ovulation rate was 59.2% (93/157), while for double ovulation, the rate was 40.8% (64/157). Single ovulation occurred with equal frequency (*p* > 0.05) on both ovaries (left ovary: 42.5%; right ovary: 32.5%). However, the incidence of bilateral double ovulation (18.3%) was significantly higher (*p* < 0.01) than that of ipsilateral double ovulation (6.7%).

A total of 124 uterine flushings were carried out during the study, of which 92 were positive (PFR: 74.2%; 92/124), and 100 embryos were recovered out of 124 estrous cycles (ERR: 80.6%; 100/124) and 157 ovulations (embryo recovery per ovulation: 63.7%; 100/157).

The embryo diameter and morphological quality score of donkey embryos are shown in Table 2. Overall, 77 of 100 embryos (77%) were classified as Grade 1 (excellent), 17 (17%) were classified as Grade 2 (good) and 6 (6%) were classified as Grade 3 (fair). The most frequent stages of development observed were early blastocyst (37%, 37/100) and expanded blastocyst (36%, 36/100), which were followed by morula (20%, 20/100) and blastocyst (7%, 7/100) stages. The embryo quality score significantly (*p* < 0.05) varied according to developmental stage and day of recovery, being lower for blastocysts or when flushed at day 8 after ovulation (Table 2). As expected, the embryo diameter was also affected (*p* < 0.001) by the developmental stage and day of flushing. The mean diameter of embryos was 179.39 ± 9.61 µm (range: 150–300 µm) for morulae, 210.81 ± 7.90 µm (range: 150–325 µm) for early blastocysts, 425.00 ± 45.32 µm (range: 275–600 µm) for blastocysts, and 1022.92 ± 125.22 µm (range: 250–3300 µm) for expanded blastocysts. Moreover, it was observed that embryos recovered at 6 days after ovulation had a diameter of 187.50 ± 15.23 µm (range: 150–300 µm); those collected on day 7 measured 236.48 ± 13.83 µm (range: 150–600 µm); while the mean diameter at 8 and 9 were 806.25 ± 83.31 µm (range: 275–2400 µm) and 2275 ± 300.14 µm (range: 1525–3300 µm), respectively (Table 2).

Presented in Table 3 are the developmental stage and embryo size for Day 6–9 embryos. At Day 6 after ovulation, embryos were mostly at the morula stage (12/13, 92.3%); meanwhile, 67.9% (36/53) of embryos recovered at Day 7 were early blastocyst stage embryos. Expanded blastocysts were recovered on Day 8 (25/28, 89.3%) and 9 (6/6, 100%). At Day 6–7, most of the embryos recovered were small (<200 µm) or medium (200–300 µm) embryos; meanwhile, large embryos (>300 µm) were recovered at Days 8 (27/28, 96.4%) and 9 after ovulation (6/6, 100%; Table 3).

Table 4 shows extrinsic factors that affect the rate of positive flushings (PFR) and the embryo recovery rate (ERR). None of the factors studied affected (*p* > 0.05) PFR or ERR; however, there was an effect of the number of flushings (1, 2, or 3) on both rates, which were significantly (*p* < 0.001) reduced in the third flushing (PFR: 15.8%; ERR: 0.16).

The intrinsic factors that affect PFR and ERR are shown in Table 5. PFR did not vary (*p* > 0.05) with any of the studied variables except the donor (*p* = 0.040). No differences between parity (*p* = 0.2610) and number of ovulations (*p* = 0.0971) were detected for ERR (Table 5). In contrast, ERR not only varies among donors (*p* < 0.05) but also among donor age groups (*p* < 0.05) and successive cycles within the donor (*p* < 0.001). ERR was higher (*p* < 0.05) in jennies of 4–9 years of age (0.94; 51/54) with respect to the other groups (≤3 years: 0.77 (26/34); ≥10 years: 0.64 (23/36)). With regard to the number of ovulations in the same donor, ERR was significantly higher (*p* < 0.001) in the first group (1–5 cycles: 1.44; 49/34) with respect to the second (6–14 cycles: 0.78; 39/50) and third (15–23 cycles: 0.30; 12/40) groups.

As shown in Table 6, PFR and ERR varied (*p* > 0.05) among jacks. Four jacks (numbers 169, 192, 2895, and 4457) that were used in 79.7% of cycles (94/118) showed good results for both rates (PFR: 70.8–100%; ERR: 0.77–1.00). The other three jacks (numbers 95, 148, and 9025) that were used in 18.6% of cycles (22/118) yield a lower PFR (50–58.3%) and ERR (0.50–0.67) than the previous group, but this was not significant statistically (*p* > 0.05). No embryos were obtained with jack number 232, although he was used only two times. 

Developmental stage, embryo quality, and diameter of the embryos recovered in this study are shown in Table 7, Table 8 and Table 9. None of the extrinsic factors studied significantly influenced (*p* < 0.05) these three variables, except for the day of flushing (6–9), which significantly influenced the developmental stage (*p* < 0.001), embryo quality (*p* < 0.05), and diameter of embryos (*p* < 0.001; Table 7). Similarly, the number of flushings (1–3) significantly influenced the diameter (*p* = 0.038) and embryo developmental stage (*p* = 0.001), whereas the developmental stage was statistically different among herds (*p* = 0.020; Table 7). 

Table 8 shows that no influence was detected (*p* > 0.05) of the intrinsic factors on developmental stage, embryo quality, and diameter of embryos, except for the donor, which affected the embryo quality (*p* = 0.004). Moreover, no differences (*p* > 0.05) were detected among jacks for these three variables (Table 9).

## 4. Discussion

Due to the similarities in the reproductive physiology between horses and donkeys, several assisted reproductive technologies (ARTs) routinely used in horses have been applied directly to donkeys. Hence, previous studies have demonstrated the suitability of mare ET techniques for collecting embryos in jennies [16]. In this line, numerous studies have been conducted to examine the factors that affect embryo recovery, quality, and diameter in mares [13,22,29]. However, in donkeys, these studies have been very scarce [17,20], and they are often performed on a limited number of animals, cycles, or embryos. Therefore, more studies are needed to optimize embryo recovery rates and maximize the success of future ET programs in donkeys.

In this study, in which 26 donor jennies and 124 cycles were used, the average ovulation rate per jenny was 1.26 ± 0.04. This finding was slightly lower than the reported average in spontaneous (1.57 ± 0.06) and prostaglandin F2 alpha (PGF2α)-induced (1.56 ± 0.10) estrus of Andalusian jennies [30]. These small differences could be explained by other factors (such as feeding management, donor age, reproductive status, season of the year, and the use of drugs to induce ovulation) that can affect the incidence of multiple ovulations, as reported in mares [22,31,32,33] and donkeys [30,34]. 

In jennies, the incidence of multiple ovulation was reported to range between 5.3% and 61% [35,36,37]. In our study, the single ovulation rate was 59.2%, while the double ovulation rate was 40.8%. Double ovulation in jennies was similar to that reported in Catalonian jennies (42.45%) [34] and in the Asinina de Miranda jennies (36.36%) [38]. However, the incidence of double ovulation in this study was lower than that reported in spontaneous (51.7%) and PGF2α-induced cycles (56.5%) in Andalusian jennies [30]. It is interesting to note that single ovulation occurred with equal frequency on both ovaries, as also reported by other authors [30,34]. Similar to that reported by Taberner et al. [34], a minimally greater frequency of ovulation for the left ovary was found (42.5% vs. 32.5%), but the difference was not significant. On the other hand, the incidence of bilateral double ovulation was significantly higher than that of ipsilateral double ovulation, which agrees with findings for mares [31] and jennies [30].

No influence of the age of the donor on the ovulation rate was observed in this study; however, the ovulation rate was numerically (1.36 ± 0.10%), but not significantly higher, in the older jennies (≥10 years old). These results are consistent with previous findings in jennies [20,30] and mares [31]. Although the reason for this fact remains still unclear, it has been suggested that increased ovarian stimulation or enhanced ovarian receptivity to that stimulation may be involved. Thus, multiovulation would be a natural strategy to ensure gestation in older females, which have a reduced ability to become pregnant [32].

The ovulation rate did not vary among years, seasons, and photoperiods, but it tended to be lower in summer (*p* = 0.095). Similar findings have been reported in previous studies [20,30]. Ginther et al. [39], in a study using different breeds and geographical latitude than our study of donkeys, observed that the incidence of multiple ovulations was not affected by the season of the year. The statistical analysis revealed significant differences among herds (*p* = 0.031), which could be explained by the individuals comprising each herd. In fact, this study pointed out the existence of significant differences among jennies (*p* = 0.01). 

In our study, in which 100 embryos were used, the overall ERR following non-surgical flushing on Days 6–9 was 80.6%, which was higher than those previously reported in different breeds of donkeys: 53.3% in jennies of unknown breed [40], 63.6% in Poitou jennies [41], 75.9% in Pantesca jennies [17], 50% in Amiata jennies [16], 52.3% in Pega jennies [42], and 40.7% in Andalusian and Zamorano-Leones jennies [20]. The EER obtained in our experiment was also higher than the rates reported in the literature for fertile mares in commercial ET programs [43]—60–77% for fresh, 44% for chilled, and 46% for frozen semen—but similar to that obtained in young fertile mares inseminated with fresh semen, 87% [44]. These results are likely due to the age of the jennies used in the study and the physical and reproductive assessment performed before, including donors in the experimental group.

It is known that the major factor affecting embryo recovery is the donors´ reproductive history. Hence, embryo recovery for old sub-fertile mares can be as low as 30–40% per cycle [45]. Other factors that affect embryo recovery include semen quality and semen type (fresh, cooled, or frozen) [45]. In our study, all donors were selected carefully, based on their reproductive history and clinical examination, and they were mated naturally with jacks of proven fertility. Although the PFR and ERR varied among jacks, seven out of eight jacks showed moderate to good results for both rates (≥50% and ≥0.50, respectively). Only one jack had low fertility (zero out of two positive flushes), but he was used only twice, which could mitigate its negative effect on average PFR and ERR. In addition, hCG was used as the ovulation inductor. Previous studies have clearly demonstrated that ovulation induction can enhance the efficiency of ARTs in domestic animal species, including the donkey [46].

The embryo morphology score is the most common method used to evaluate embryo quality [27]. In line with previous findings [16,17,20,42], 94% of the recovered embryos had a quality grade of excellent (Grade 1) or good (Grade 2). 

In our experiment, none of the extrinsic factors analyzed (year of the study, season of the year, photoperiod, herd or day, of flushing) affected significantly PFR or ERR. Consistent with previous findings [17], the ERR obtained in the first year (0.73) was numerically lower than that obtained in the second (0.86) and third (0.82) year, which could be explained by the inexperience with this technology (i.e., ET) in donkeys. The absence of a photoperiod influence on embryo recovery has been previously described in donkeys [17,20]. The study carried out in Pantesca donkeys [17] also noted that the time of the year did not affect PFR and ERR. Considering these results, we could state that seasonality has little impact on reproductive performance of Andalusian jennies, which can get pregnant naturally all year round, as previously reported for other donkey breeds [17]. This fact brings the possibility of applying ET in Andalusian donkeys along the year.

In donkeys, the influence of the day of flushing on ERR has not been well established in the literature [17,20]. Under our experimental conditions, PFR and ERR were not different among Days 6, 7, 8, and 9. Similar findings have been reported in mares [22,47]. However, embryo diameter and developmental stage varied widely depending on the day of recovery, which is consistent with previous studies [16,41]. In line with these findings, poor-quality embryos were collected at Day 8, and ERR tended to be lower (*p* = 0.08), flushing the uterus 9 days after ovulation. Taken all together, our results emphasize the importance of collecting Day 6 to 7 donkey embryos, smaller than 300 µm in diameter and with good morphological score, to ensure vitrification success, as has been previously suggested [19]. 

From a practical point of view, another interesting observation was the effect of the number of flushings (1, 2, or 3) on both rates (PFR and ERR), which were significantly reduced in the third flushing (15.8% and 0.16, respectively), indicating that the majority of embryo collections require one or two maximum flushings. In addition, embryo diameter and developmental stage varied among flushings, with larger and older embryos in the first flushing. Thus, our results could suggest that the larger diameter of the older embryos could facilitate their recovery [17].

The embryos recovered in herd 1 were in earlier developmental stages than in the other herds. These results were probably due to the fact that all the embryo rerecovered in this herd (5 embryos) were flushed 6–7 days after ovulation, while in herds 2 (56 embryos) and 3 (39 embryos), flushes were carried out from Day 6 to 9 after ovulation, thus increasing the average diameter and developmental stage of the recovered embryos. 

Regarding the intrinsic factors, PFR did not vary with any of the studied variables (i.e., donor age, parity, successive cycle within donor, and number of ovulations) except for the donor jenny. In contrast, ERR not only varied among donors but also among donor age categories, showing higher values in jennies of 4–9 years of age (0.94) compared with all the other categories of age. Moreover, both PFR and ERR were not different between younger (≤3 years old) and older (≥10 years old) jennies. Our findings are in contrast with the results of previous studies [17,20], which reported no effect of donor age and donor jenny on the aforementioned rates. However, the effect on ERR of donor age and donor mare have been reported by many authors [13,22,48,49], in which old age (>15 years old) and a history of sub-fertility were related to a lower ERR. Our results could suggest that embryo donors between 4 and 9 years are the best to be used in an ET program. 

The effect of repeated uterine flushings has been previously described in mares [50], which was associated with increased chronic inflammation of the uterus. Although previous studies failed to observe this negative effect in donkeys [17], in our experiment, the ERR on successive cycles from 1 to 5 was higher (1.44) than in attempts from 6 to 14 (0.78) and from 15 to 23 (0.30). The differences observed between studies may be explained by different experimental conditions. Therefore, in our study, young (≤3 years), mature (4–9 years), and old (≥10 years) donor jennies were employed during the entire period of the study, while only young jennies (2–5 years old) were used in this previous work [17]. Moreover, a higher number of donors (10, 8, and 6, respectively) and cycles (63, 45, and 16, respectively) were used in each group.

It has been previously described that the occurrence of multiple ovulations enhances ERR in mares [22,51] and donkeys [17,20], but this effect was not shown in our study. However, despite the absence of statistical significance, ERR after single ovulation tended to be lower than that obtained after double ovulation (0.77 vs. 0.91; *p* = 0.0971). It has been also reported that ipsilateral double ovulations resulted in a lower ERR than bilateral double ovulations [52], which could be caused by interference between two or more simultaneous ovulations in the limited space of the ovulation fossa [23,53]. In the present study, the incidence of ipsilateral double ovulations was only 6.7%, and no significant difference in ERR was observed between bilateral and ipsilateral ovulations (1.62% vs. 1.88%; *p* = 0.196).

Conversely, the number of ovulations in the same donor influenced ERR, being significantly higher in the first group (1–5 cycles: 1.44; 49/34), but no effect on PFR was observed. These results partially agree with the findings of Camillo et al. [17], who observed a significant effect on both rates. Finally, we observed that the parity of the donors did not have an effect on PFR and ERR. In cattle, the parity of recipients does not affect pregnancy rates following the transfer of fresh and frozen embryos [54]. However, to our knowledge, no data are available for mare and jenny donors.

## 5. Conclusions

Based on our results, we can conclude that the donor jenny was the main factor that affects the rate of positive flushings and recovery rates as well as the embryo quality. Other factors that affected embryo recovery rate were the number of flushings, donor age, successive cycle within donor, and jack. Meanwhile, the rate of positive flushings was affected by the number of flushings and the jack. From a practical point of view, these findings could indicate that the majority of embryo collections require one or two maximum flushings per cycle. Moreover, the negative effect of repeated uterine flushings on embryo recovery rate was proven, being lower after six consecutive cycles. On the other hand, the day of flushing had a significant effect on embryo quality and diameter, which emphasizes the importance of collecting Day 6 to 7 donkey embryos, with good morphological score and smaller than 300 µm in diameter, if embryos are going to be cryopreserved.

## Figures and Tables

**Table 1:** Ovulation rate (mean ± SEM) in 124 cycles of 26 donor jennies according to donor age categories (≤3, 4–9 or ≥10 years old), parity (nulliparous or multiparous), year of the study (first, second or third), season of the year (spring, summer, autumn, or winter), photoperiod (positive or negative), and herd (1, 2, or 3).

Variable	No. of Cycles	Ovulation Rate	*p*-Values
Donor age			
≤3 years	34	1.24 ± 0.07	0.433
4–9 years	65	1.23 ± 0.05	
≥10 years	25	1.36 ± 0.10	
Parity			
Nulliparous	35	1.26 ± 0.08	0.988
Multiparous	89	1.26 ± 0.05	
Year			
First	33	1.21 ± 0.07	0.073
Second	42	1.17 ± 0.06	
Third	49	1.37 ± 0.07	
Season			
Spring	44	1.18 ± 0.06	0.095
Summer	12	1.08 ± 0.08	
Autumn	52	1.37 ± 0.07	
Winter	16	1.25 ± 0.11	
Photoperiod			
Positive	66	1.20 ± 0.05	0.099
Negative	58	1.33 ± 0.06	
Herd			
1	6	1.33 ± 0.21 ^ab^	0.031
2	71	1.17 ± 0.05 ^b^	
3	47	1.38 ± 0.07 ^a^	
Total	124	1.26 ± 0.04	

^a,b^ Values with different superscript differ significantly.

**Table 2:** Diameters (mean ± SEM) and morphological quality score (1–4) of donkey embryos collected at Days 6 to 9 after ovulation according to their developmental stage (morula, early blastocyst, blastocyst, or expanded blastocyst) and day of recovery (6, 7, 8, or 9).

Variable	Grade	Diameter (µm)	No. (%)	Embryo Quality at Collection		
				Grade 1	Grade 2	Grade 3
Developmental stages						
Morula	1.50 ± 0.15 ^b^	179.39 ± 9.61 ^a^	20 (20%)	12 (60%) ^a^	6 (30%)	2 (10%)
Early blastocyst	1.38 ± 0.11 ^ab^	210.81 ± 7.90 ^a^	37 (37%)	26 (70.3%) ^a^	8 (21.6%)	3 (8.1%)
Blastocyst	1.00 ± 0.00 ^a^	425.00 ± 45.32 ^a^	7 (7%)	7 (100%) ^b^	0 (0%)	0 (0%)
Expanded blastocyst	1.14 ± 0.07 ^ab^	1022.92 ± 125.22 ^b^	36 (36%)	32 (88.9%) ^b^	3 (8.3%)	1 (2.8%)
Day of recovery						
6	1.46 ± 0.22 ^b^	187.50 ± 15.23 ^a^	13 (13%)	9 (69.2%) ^a^	2 (15.4%) ^b^	2 (15.4%) ^b^
7	1.38 ± 0.08 ^ab^	236.48 ± 13.83 ^a^	53 (53%)	36 (67.9%) ^a^	14 (26.4%) ^b^	3 (5.7%) ^a^
8	1.04 ± 0.04 ^a^	806.25 ± 83.31 ^b^	28 (28%)	27 (96.4%) ^b^	1 (3.6%) ^b^	0 (0%) ^a^
9	1.33 ± 0.33 ^ab^	2275.00 ± 300.14 ^c^	6 (6%)	5 (83.3%) ^a^	0 (0%) ^a^	1 (16.7%) ^b^
Total	1.29 ± 0.06	515.24 ± 59.95	100 (100%)	77 (77%)	17 (17%)	6 (6%)

^a–c^ Values with different superscript differ significantly (*p* < 0.05).

**Table 3:** Developmental stage (morula, early blastocyst, blastocyst, or expanded blastocyst) and embryo size (<200 µm, 200–300 µm or >300 µm) of donkey embryos collected at Day 6 to 9 after ovulation.

Day of Recovery	No. (%)	Developmental Stage				Embryo Diameter		
		Morula	Early Blastocyst	Blastocyst	Expanded Blastocyst	<200 µm	200–300 µm	>300 µm
6	13 (13%)	12 (92.3%) ^a^	1 (7.7%) ^b^	0 (0%) ^b^	0 (0%) ^b^	9 (69.2%) ^a^	4 (30.8%) ^b^	0 (0%) ^c^
7	53 (53%)	8 (15.1%) ^b^	36 (67.9%) ^a^	4 (7.5%) ^b^	5 (9.4%) ^b^	24 (45.3%) ^a^	19 (35.8%) ^a^	10 (18.9%) ^b^
8	28 (28%)	0 (0%) ^b^	0 (0%) ^b^	3 (10.7%) ^b^	25 (89.3%) ^a^	0 (0%) ^b^	1 (3.6%) ^b^	27 (96.4%) ^a^
9	6 (6%)	0 (0%) ^b^	0 (0%) ^b^	0 (0%) ^b^	6 (100%) ^a^	0 (0%) ^b^	0 (0%) ^b^	6 (100%) ^a^
Total	100 (100%)	20 (20%)	37 (37%)	7 (7%)	36 (36%)	43 (43%)	14 (14%)	43 (43%)

^a–c^ Values with different superscript differ significantly (*p* < 0.05).

**Table 4:** Extrinsic factors affecting the rate of positive flushings and embryo recovery rate in Andalusian donkeys.

Factor	Positive Flushings	*p*-Value	Embryo Recovery Rate	*p*-Value
Year				
First	21/33 (63.6%)	NS	24/33 (0.73)	NS
Second	33/42 (78.6%)		36/42 (0.86)	
Third	38/49 (77.6%)		40/49 (0.82)	
Season				
Spring	33/44 (75.0%)	NS	34/44 (0.77)	NS
Summer	10/12 (83.3%)		10/12 (0.83)	
Autumn	36/52 (69.2%)		42/53 (0.79)	
Winter	13/16 (81.3%)		14/15 (0.93)	
Photoperiod				
Positive	50/66 (75.8%)	NS	53/66 (0.80)	NS
Negative	42/58 (72.4%)		47/58 (0.81)	
Herd				
1	4/6 (66.7%)	NS	5/6 (0.83)	NS
2	51/71 (71.8%)		56/71 (0.79)	
3	37/47 (78.7%)		39/47 (0.83)	
Day of flushing				
6	12/16 (75.0%)	NS	13/16 (0.81)	NS
7	49/68 (72.1%)		53/68 (0.80)	
8	25/31 (80.6%)		28/31 (0.90)	
9	6/9 (66.7%)		6/9 (0.67)	
No. of flushings				
1	66/66 (100%) ^a^	0.001	73/66 (1.11) ^a^	0.001
2	20/20 (100%) ^a^		21/20 (1.05) ^a^	
3	6/38 (15.8%) ^b^		6/38 (0.16) ^b^	
Total	92/124 (74.2%)		100/124 (0.81)	

^a,b^ Values with different superscript differ significantly; NS, not significant.

**Table 5:** Intrinsic factors affecting the rate of positive flushings and embryo recovery rate in Andalusian donkeys.

Factor	Positive Flushings	*p*–Value	Embryo Recovery Rate	*p*–Value
Donor				
60	0/1 (0%) ^b^	0.040	0/1 (0.00) ^b^	0.011
64	1/1 (100%) ^a^		1/1 (1.00) ^a^	
142	9/10 (90%) ^a^		9/10 (0.90) ^a^	
167	10/17(58.8%) ^ab^		11/17 (0.65) ^a^	
193	4/5(80%) ^a^		4/5(0.80) ^a^	
1159	1/2 (50%) ^ab^		1/2 (0.50) ^ab^	
1161	14/18 (77.8%) ^a^		15/18 (0.83) ^a^	
1220	1/1 (100%) ^a^		1/1 (1.00) ^a^	
1372	1/1 (100%) ^a^		1/1 (1.00) ^a^	
1826	0/1 (0%) ^b^		0/1 (0.00) ^b^	
2089	1/1 (100%) ^a^		2/1 (2.00) ^a^	
2339	1/1 (100%) ^a^		1/1 (1.00) ^a^	
2629	4/8 (50%) ^ab^		4/8 (0.50) ^ab^	
3223	6/6 (100%) ^a^		6/6 (1.00) ^a^	
3977	1/1 (100%) ^a^		1/1 (1.00) ^a^	
4103	12/13 (92.3%) ^a^		12/13 (0.92) ^a^	
5372	0/1 (0%) ^b^		0/1 (0.00) ^b^	
6069	1/1 (100%) ^a^		1/1 (1.00) ^a^	
6517	2/2 (100%) ^a^		2/2 (1.00) ^a^	
7148	12/15 (80%) ^a^		14/15 (0.93) ^a^	
7171	2/6 (33.3%) ^ab^		2/6 (0.33) ^b^	
7590	0/2 (0%) ^b^		0/2 (0.00) ^b^	
8310	2/2 (100%) ^a^		3/2 (1.50) ^a^	
8311	6/6 (100%) ^a^		8/6 (1.33) ^a^	
9695	1/2 (50%) ^ab^		1/2 (0.50) ^ab^	
Donor age				
≤3 years	25/34 (73.5%)	NS	26/34 (0.77) ^b^	0.013
4–9 years	48/65 (73.8%)		51/54 (0.94) ^a^	
≥10 years	19/25 (76%)		23/36 (0.64) ^b^	
Parity				
Nulliparous	25/35 (71.4%)	NS	26/35 (0.74)	NS
Multiparous	67/89 (75.3%)		74/89 (0.83)	
Successive cycle within donor				
1–5	44/63 (69.8%)	NS	49/34 (1.44) ^a^	0.001
6–14	36/45 (80%)		39/50 (0.78) ^b^	
15–23	12/16 (75%)		12/40 (0.30) ^c^	
No. of ovulations				
Single	70/92 (76.1%)	NS	71/92 (0.77)	NS
Double	22/32 (68.8%)		29/32 (0.91)	
Total	92/124 (74.2%)		100/124 (0.81)	

^a–c^ Values with different superscript differ significantly; NS, not significant.

**Table 6:** Variation in the rate of positive flushings and embryo recovery rate between Andalusian jacks.

Jack	Mated Donors	Positive Flushings	*p*-Value	Embryo Recovery Rate	*p*-Value
95	12	7/12 (58.3%) ^ab^	0.0253	8/12 (0.67) ^ab^	0.0253
169	36	27/36 (75%) ^a^		29/36 (0.81) ^a^	
192	3	3/3 (100%) ^a^		3/3 (1.00) ^a^	
232	2	0/2 (0%) ^b^		0/2 (0.00) ^b^	
1481	2	1/2 (50%) ^ab^		1/2 (0.50) ^ab^	
2895	7	6/7 (85.7%) ^a^		6/7 (0.86) ^a^	
4457	48	34/48 (70.8%) ^a^		37/48 (0.77) ^a^	
9025	8	4/8 (50%) ^ab^		4/8 (0.50) ^ab^	
Total	118 *	82/118 (69.5%)		88/118 (0.75)	

^a,b^ Values with different superscript differ significantly; * Missing data (*n* = 6).

**Table 7:** Extrinsic factors affecting the developmental stage, embryo quality, and diameter of embryos in Andalusian donkeys.

Factor	No.	Developmental Stage	*p*-Value	Embryo Grade	*p*-Value	Embryo Diameter	*p*-Value
Year							
First	24	2.25 ± 0.24	NS	1.25 ± 0.11	NS	548.91 ± 140.10	NS
Second	36	2.69 ± 0.20		1.28 ± 0.10		637.50 ± 124.22	
Third	40	2.70 ± 0.18		1.33 ± 0.09		385.83 ± 52.24	
Season							
Spring	34	2.74 ± 0.20	NS	1.26 ± 0.09	NS	660.29 ± 142.21	NS
Summer	10	3.20 ± 0.33		1.10 ± 0.10		602.50 ± 176.48	
Autumn	42	2.38 ± 0.18		1.40 ± 0.10		410.91 ± 62.66	
Winter	14	2.43 ± 0.33		1.14 ± 0.14		405.77 ± 87.90	
Photoperiod							
Positive	53	2.66 ± 0.16	NS	1.25 ± 0.07	NS	619.87 ± 100.27	NS
Negative	47	2.51 ± 0.17		1.34 ± 0.09		399.47 ± 56.92	
Herd							
1	5	1.20 ± 0.20 ^a^	0.020	1.60 ± 0.40	NS	270.83 ± 30.90	NS
2	56	2.61 ± 0.16 ^b^		1.25 ± 0.07		619.20 ± 97.36	
3	39	2.74 ± 0.12 ^b^		1.31 ± 0.09		391.03 ± 53.33	
Day of flushing							
6	13	1.08 ± 0.08 ^a^	0.000	1.46 ± 0.22 ^b^	0.045	187.50 ± 15.23 ^a^	0.000
7	53	2.11 ± 0.11 ^b^		1.38 ± 0.08 ^b^		236.48 ± 13.83 ^a^	
8	28	3.89 ± 0.06 ^c^		1.04 ± 0.04 ^a^		806.25 ± 83.31 ^b^	
9	6	4.00 ± 0.00 ^c^		1.33 ± 0.33 ^ab^		2275.00 ± 300.14 ^c^	
No. of flushings							
1	73	2.84 ± 0.14 ^c^	0.001	1.23 ± 0.06	NS	604.79 ± 78.25 ^b^	0.038
2	21	2.05 ± 0.22 ^ab^		1.48 ± 0.15		290.42 ± 32.19 ^a^	
3	6	1.50 ± 0.22 ^a^		1.33 ± 0.21		175.00 ± 9.13 ^a^	
Total	100	2.59 ± 0.14		1.29 ± 0.06		515.24 ± 59.95	

^a–c^ Values with different superscript differ significantly; NS, not significant.

**Table 8:** Intrinsic factors affecting the developmental stage, embryo quality, and diameter of embryos in Andalusian donkeys.

Factor	No.	Developmental Stage	*p*-Value	Embryo Grade	*p*-Value	Embryo Diameter	*p*-Value
Donor							
60	*	-	NS	-	0.004	-	NS
64	1	1.00 ± -		1.00 ± -		-	
142	9	2.89 ± 0.39		1.11 ± 0.11 ^a^		631.11 ± 244.42	
167	11	2.36 ± 0.34		1.36 ± 0.15 ^ab^		279.55 ± 55.17	
193	4	3.00 ± 0.58		1.25 ± 0.25 ^ab^		368.75 ± 110.10	
1159	1	2.00 ± -		1.00 ± -		200.00 ± -	
1161	15	1.50 ± 0.22		1.20 ± 0.15 ^ab^		405.00 ± 101.07	
1220	1	1.00 ± -		1.00 ± -		175.00 ± -	
1372	1	1.00 ± -		2.00 ± -		175.00 ± -	
1826	*	-		-		-	
2089	2	1.00 ± 0.00		2.00 ± 1.00 ^b^		287.50 ± 12.50	
2339	1	1.00 ± -		1.00 ± -		175.00 ± -	
2629	4	3.50 ± 0.50		1.00 ± 0.00 ^a^		1143.75 ± 722.51	
3223	6	2.67 ± 0.56		1.50 ± 0.34 ^ab^		954.17 ± 440.29	
3977	1	4.00 ± -		1.00 ± -		1625.00 ± -	
4103	12	3.50 ± 0.26		1.17 ± 0.11 ^a^		662.50 ± 181.36	
5372	*	-		-		-	
6069	1	1.00 ± -		2.00 ± -		183.33 ± -	
6517	2	2.00 ± 0.00		3.00 ± 0.00 ^c^		325.00 ± 0.00	
7148	14	2.21 ± 0.33		1.43 ± 0.17 ^ab^		360.71 ± 92.94	
7171	2	2.00 ± 1.00		1.00 ± 0.00 ^a^		262.50 ± 112.50	
7590	*	-		-		-	
8310	3	3.00 ± 1.00		1.00 ± 0.00 ^a^		725.00 ± 291.91	
8311	8	2.63 ± 0.42		1.13 ± 0.13 ^a^		571.88 ± 222.88	
9695	1	2.00 ± -		1.00 ± -		325.00 ± -	
Donor age							
≤3 years	26	2.58 ± 0.22	NS	1.27 ± 0.09	NS	414.74 ± 91.94	NS
4–9 years	51	2.67 ± 0.17		1.29 ± 0.09		579.50 ± 96.80	
≥10 years	23	2.43 ± 0.26		1.30 ± 0.12		489.13 ± 108.25	
Parity							
Nulliparous	26	2.58 ± 0.22	NS	1.27 ± 0.09	NS	414.74 ± 91.94	NS
Multiparous	74	2.59 ± 0.14		1.30 ± 0.07		551.03 ± 74.30	
Successive cycle within donor							
1–5	49	2.41 ± 0.17	NS	1.35 ± 0.09	NS	556.42 ± 93.53	NS
6–14	39	2.72 ± 0.19		1.26 ± 0.09		534.62 ± 97.97	
15–23	12	2.92 ± 0.29		1.17 ± 0.11		287.50 ± 37.75	
No. of ovulations							
Single	71	2.61 ± 0.14	NS	1.25 ± 0.06	NS	538.33 ± 79.70	NS
Double	29	2.55 ± 0.23		1.38 ± 0.13		459.48 ± 70.70	
Total	100	2.59 ± 0.14		1.29 ± 0.06		515.24 ± 59.95	

^a–c^ Values with different superscript differ significantly; NS, not significant; * *Missing data*.

**Table 9:** Variation in developmental stage, embryo quality, and diameter of embryos between Andalusian jacks.

Jack	Mated Donors	Developmental Stage	*p*-Value	Embryo Grade	*p*-Value	Embryo Diameter	*p*-Value
95	8	3.00 ± 0.38	NS	1.25 ± 0.16	NS	475.00 ± 140.71	NS
169	29	3.03 ± 0.21		1.24 ± 0.08		447.41 ± 67.59	
192	3	2.33 ± 0.33		1.67 ± 0.67		258.33 ± 44.10	
1481	1	1.00 ± -		2.00 ± -		183.33 ± -	
2895	6	1.83 ± 0.48		1.50 ± 0.22		366.67 ± 177.09	
4457	37	2.51 ± 0.19		1.27 ± 0.10		629.05 ± 136.72	
9025	8	2.25 ± 0.75		1.00 ± 0.00		681.25 ± 412.36	
Total	88 *	2.65 ± 0.12		1.28 ± 0.06		521.97 ± 66.52	

NS, not significant; * Missing data (*n* = 12).
